# Dual disease co-expression analysis reveals potential roles of estrogen-related genes in postmenopausal osteoporosis and Parkinson’s disease

**DOI:** 10.3389/fgene.2024.1518471

**Published:** 2025-01-07

**Authors:** Dongdong Yu, Jian Kang, Chengguo Ju, Qingyan Wang, Ye Qiao, Long Qiao, Dongxiang Yang

**Affiliations:** ^1^ First Clinical School, Liaoning University of Traditional Chinese Medicine, Shenyang, China; ^2^ Orthopedics and Traumatology, Affiliated Hospital of Liaoning University of Traditional Chinese Medicine, Shenyang, China; ^3^ Graduate School, Liaoning University of Traditional Chinese Medicine, Shenyang, China

**Keywords:** postmenopausal osteoporosis, Parkinson disease, differentially expressed genes, estrogen, Bayesian colocalization analysis

## Abstract

**Introduction:**

The deficiency of estrogen correlates with a range of diseases, notably Postmenopausal osteoporosis (PMO) and Parkinson’s disease (PD). There is a possibility that PMO and PD may share underlying molecular mechanisms that are pivotal in their development and progression. The objective of this study was to identify critical genes and potential mechanisms associated with PMO by examining co-expressed genes linked to PD.

**Methods:**

Initially, pertinent data concerning PMO and PD were obtained from the GWAS database, followed by conducting a Bayesian colocalization analysis. Subsequently, co-expressed genes from the PMO dataset (GSE35956) and the PD dataset (GSE20164) were identified and cross-referenced with estrogen-related genes (ERGs). Differentially expressed genes (DEGs) among PMO, PD, and ERGs were subjected to an array of bioinformatics analyses, including Kyoto Encyclopedia of Genes and Genomes (KEGG) and Gene Ontology (GO) enrichment analyses, in addition to protein-protein interaction (PPI) network analysis. The study also involved constructing TF-gene interactions, TF-microRNA coregulatory networks, interactions of hub genes with diseases, and validation through quantitative reverse transcription polymerase chain reaction (qRT-PCR).

**Results:**

The colocalization analysis uncovered shared genetic variants between PD and osteoporosis, with a posterior probability of colocalization (PPH4) measured at 0.967. Notably, rs3796661 was recognized as a shared genetic variant. A total of 11 genes were classified as DEGs across PMO, PD, and ERGs. Five principal KEGG pathways were identified, which include the p53 signaling pathway, TGF-beta signaling pathway, cell cycle, FoxO signaling pathway, and cellular senescence. Additionally, three hub genes—WT1, CCNB1, and SMAD7—were selected from the PPI network utilizing Cytoscape software. These three hub genes, which possess significant diagnostic value for PMO and PD, were further validated using GEO datasets. Interactions between transcription factors and genes, as well as between microRNAs and hub genes, were established. Ultimately, the expression trends of the identified hub genes were confirmed through qRT-PCR validation.

**Conclusions:**

This study is anticipated to offer innovative approaches for identifying potential biomarkers and important therapeutic targets for both PMO and PD.

## 1 Introduction

Postmenopausal osteoporosis (PMO) represents a prevalent metabolic bone disorder characterized by a decline in bone mass and an increase in fragility, which significantly heightens the likelihood of fractures (Xu et al., 2017). The etiology of PMO is multifaceted, with estrogen deficiency being the primary contributor, resulting in bone loss among postmenopausal women (Xiao et al., 2024). In cases of PMO, diminished estradiol (E2) synthesis disrupts the equilibrium of bone metabolism. This alteration affects the signaling pathways of estrogen receptors (ER), leading to a reduction in osteoblast function and an enhancement in osteoclast activity (Shih et al., 2019). The loss of estrogen instigates inflammatory responses within the bone microenvironment, culminating in accelerated bone loss and osteoporosis in nearly half of postmenopausal women (Zhang et al., 2020). Consequently, PMO emerges as a critical public health concern, underscoring the necessity for effective and safe therapeutic interventions. A thorough understanding of the underlying mechanisms contributing to PMO is imperative for the development of both preventive and therapeutic pharmacological agents.

Parkinson’s disease (PD) is a widespread neurological condition, with estrogen deficiency recognized as a contributing risk factor (Nathan et al., 2012). The absence of estrogen has been shown to exacerbate PD symptoms in postmenopausal females. Research indicates that estrogen therapy can mitigate early symptoms associated with PD in women (Parkinson Study Group POETRY Investigators, 2011).

Although PMO is classified as a metabolic bone disease and PD as a neurodegenerative disorder, both conditions are intricately linked through the commonality of estrogen deficiency. Despite their differing phenotypic presentations and pathological mechanisms, PMO and PD may share analogous molecular pathways that involve similar regulatory genes. The interplay of these shared mechanisms may significantly influence the pathogenesis and progression of PMO.

**FIGURE 1:**
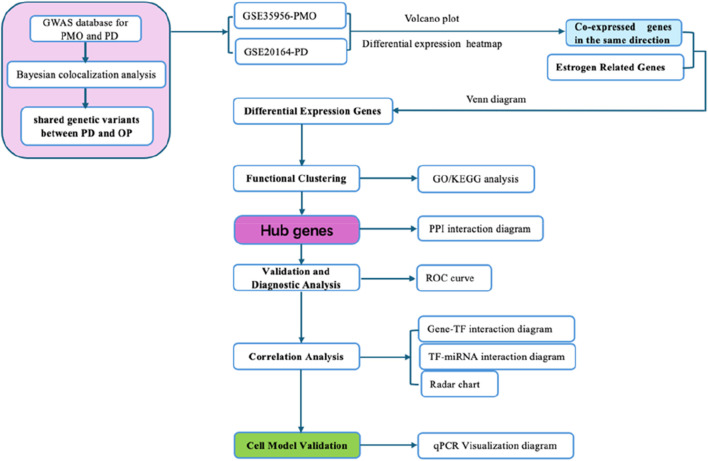
Flow chart of this study.

Co-expression gene analysis examines gene expression datasets derived from diverse samples with the objective of identifying genes that exhibit co-expression or regulation across various diseases and biological contexts. This analytical approach facilitates the understanding of how genes function synergistically and the regulatory frameworks they share. By scrutinizing the expression profiles of estrogen-related genes in both PMO and PD, we can identify critical genes and pathways uniquely associated with PMO. Such analyses may uncover potential interconnections between PMO and PD, thereby paving the way for novel research avenues and therapeutic strategies aimed at managing PMO (Figure 1).

## 2 Materials and methods

### 2.1 Bayesian colocalization analysis

The pooled dataset derived from Genome-Wide Association Studies (GWAS) concerning Osteoporosis (OP) encompasses information on 9,794 cases of individuals of Japanese ancestry alongside 168,932 controls (https://pheweb.jp/). The diagnoses of OP in this investigation were sourced from hospital records and categorized in accordance with the International Classification of Diseases and Related Health Problems (ICD) 10 coding framework (Sakaue et al., 2021). Furthermore, the GWAS pooled dataset for PD comprises data from 1,050 cases identified as Korean patients and 5,000 controls (https://www.ebi.ac.uk/gwas/studies/GCST90278092). In this study, sporadic PD diagnoses were established by specialists in movement disorders, adhering to the criteria set forth by the United Kingdom Parkinson’s Disease Brain Bank. Baseline demographic information was meticulously gathered, which included variables such as the age at which samples were collected, the age at which PD symptoms first manifested, sex, and a family history pertaining to Parkinson’s disease. The onset of symptoms was specifically defined as the moment when any primary movement-related symptoms—such as resting tremor, muscle stiffness, bradykinesia, stooped posture, or postural instability—were first observed by the patient or caregiver (Park K. W. et al., 2023).

Bayesian colocalization analysis serves to evaluate the likelihood of one or more genetic variants causally influencing two correlated traits. This analytical approach considers five mutually exclusive hypotheses: (1) H0: there is no association between the genetic variant and either trait; (2) H1: there is an association with trait 1 exclusively; (3) H2: there is an association with trait 2 solely; (4) H3: there are associations with both traits through two distinct causal variants; and (5) H4: there is an association with both traits via a shared causal variant. A posterior probability for hypothesis H4 (PPH4) that surpasses 75% is regarded as indicative of a shared causal variant that affects both traits (Giambartolomei et al., 2014).

### 2.2 Selection and processing of datasets

In this investigation, we examined frequently differentially expressed genes in PMO and PD by utilizing datasets sourced from the Gene Expression Omnibus (GEO) database. Specifically, the analysis focused on the PMO dataset GSE35956, which comprised 5 PMO samples alongside 3 normal samples, and the PD dataset GSE20164, which included 2 PD samples in conjunction with 4 normal samples. Furthermore, we retrieved information regarding estrogen-related genes (ERGs) from the GeneCards database. (https://www.genecards.org).

### 2.3 Identification of differentially expressed genes

Differentially expressed genes (DEGs) were identified through the examination of the GSE35956 and GSE20164 datasets, employing the thresholds of |log2 fold change (FC)| greater than 1 and a *p*-value of less than 0.05. The visual representation of these DEGs was accomplished using volcano plots and heatmaps, which effectively illustrated the variations in gene expression (Tian et al., 2023). Furthermore, we performed an intersection of the DEGs from both datasets with ERGs by utilizing the Venn online platform to generate Venn diagrams. This methodology enabled us to pinpoint the common DEGs present across the three datasets. The identified shared DEGs were subsequently preserved for additional analysis. The analytical tools utilized in this study were provided by Xiantao Academic (https://www.xiantaozi.com).

### 2.4 Functional annotation and pathway enrichment analysis of DEGs

We conducted functional annotation and pathway enrichment analysis to clarify the biological significance of the DEGs and their potential roles in various cellular processes. We used the Sangerbox platform (http://vip.sangerbox.com/login.html) to perform statistical analysis and visualize gene functions. We conducted Gene Ontology (GO) enrichment analysis to classify the DEGs into three categories: biological processes (BP), molecular functions (MF), and cellular components (CC). We also performed Kyoto Encyclopedia of Genes and Genomes (KEGG) pathway analysis to identify pathways significantly enriched with DEGs, which are crucial for understanding the specific biological phenomena being studied. We selected the enriched GO terms and KEGG pathways based on a *p*-value threshold of less than 0.05 to ensure their statistical significance.

### 2.5 Construction of PPI networks and identification of hub genes

The construction of protein-protein interaction (PPI) networks and the identification of hub genes are crucial for elucidating the intricate relationships and functional contributions of proteins within biological systems. Initially, we inputted the 11 DEGs into the STRING database (http://string-db.org/) to retrieve interaction data. The minimum threshold for the interaction score was established at 0.15, and there were no restrictions placed on the number of interactors presented. Subsequently, we utilized Cytoscape software to assemble and visualize the PPI network. To pinpoint the most significant genes, we employed the CytoHubba plugin, which facilitated the identification of hub genes based on metrics such as Degree, Closeness, and Betweenness. Genes exhibiting values exceeding the median were designated as hub genes.

### 2.6 Gene-TF and Gene-miRNA networks of hub genes

In order to develop the Gene-TF and Gene-miRNA networks corresponding to the identified hub genes, we utilized a variety of bioinformatics resources and tools. Initially, the three hub genes of interest were input into the NetworkAnalyst platform (https://www.networkanalyst.ca). To construct the Gene-TF network, we identified transcription factor targets leveraging information from the JASPAR database, which provides profiles of TF binding sites. Conversely, for the Gene-miRNA network, we sourced experimentally validated interaction data between miRNAs and genes from miRTarBase (Chen et al., 2023).

### 2.7 Hub gene interaction with diseases

In this study, we investigated the relationships among hub genes and a range of musculoskeletal and neurological disorders by utilizing data sourced from the Comparative Toxicogenomics Database (CTD). We extracted inference scores pertaining to hub genes linked with various conditions in PMO, bone resorption (BR), musculoskeletal diseases (MD), metabolic bone diseases (MBD), and developmental bone diseases (DBD). Furthermore, we analyzed conditions related to PD, which included PD itself, memory disorders (MD), neurobehavioral manifestations (NM), Alzheimer’s disease (AD), and stroke. The interactions between these hub genes and the specified conditions were examined through the CTD, and we employed a radar chart for the visualization of inference scores, thereby effectively demonstrating the correlations between the genes and the associated diseases.

### 2.8 Validation by qRT-PCR

The MC3T3-E1 cell line was procured from Procell (Hubei, Wuhan) and maintained in α-MEM supplemented with 10% fetal bovine serum (FBS) and 1% penicillin-streptomycin (Table 1). These cells were cultured at 37°C within a humidified environment containing 5% CO_2_ (Sun et al., 2014). To induce oxidative stress, a concentration of 250 mM hydrogen peroxide was applied to the cells for a duration of 6 h. In contrast, control cells were subjected to identical incubation conditions without the introduction of hydrogen peroxide. Bone marrow stromal cells (BMSCs) were also sourced from Procell (Hubei, Wuhan) and were cultivated in Dulbecco’s Modified Eagle Medium (DMEM) with the same supplementation of 10% FBS and 1% penicillin-streptomycin. These cells, too, were incubated at 37°C in a humidified atmosphere containing 5% CO_2_. To mimic cellular aging, BMSCs were treated with 100 μM hydrogen peroxide for 4 h (Li et al., 2017; Li et al., 2022).

**TABLE 1:** The primers for BMSCs, MC3T3-E1 cells and PC12.

Gene name	Primer	Primer sequence (5′-3′)
SMAD7	Foward	5′-CAG​CTC​AAT​TCG​GAC​AAC​AAG​A-3′
	Reverse	5′-GTA​CAC​CCA​CAC​ACC​ATC​CAC-3′
CCNB1	Foward	AAGAGCTTT AAACTTTGGTCT GGG
	Reverse	CT TTG​TAA​GTC​CTT​GAT​TTA​CCA​TG
WT1	Foward	GAT​AAC​CAC​ACA​ACG​CCC​ATC
	Reverse	CAC​ACG​TCG​CAC​ATC​CTG​AAT
GAPDH	Foward	CCT​CGT​CCC​GTA​GAC​AAA​ATG
	Reverse	TGA​GGT​CAA​TGA​AGG​GGT​CGT

PC12 cells were maintained in RPMI 1640 medium enriched with glutamine (Gibco, United States) and supplemented with 10% fetal bovine serum (Gibco, Inc.). These cells were incubated at 37°C in a humidified environment with 5% CO_2_. To establish a hypoxic model, the PC12 cells were initially cultured at 37°C with 5% CO_2_ for a duration of 24 h. Subsequently, the cells were transferred to a hypoxic chamber (Billups-Rothenburg, United States) and subjected to an oxygen-deficient gas mixture comprising 1% O_2_, 5% CO_2_, and 92% N_2_ for a period of 6 h. Following this exposure, the cells were returned to standard laboratory conditions for an additional 24 h before being collected for subsequent analysis. Control cells were maintained under standard conditions to serve as a reference for comparison (Chen et al., 2016).

The qRT-PCR was conducted by Servicbio company (Wuhan, China). The total RNA was isolated from the cells utilizing Trizol reagent (Invitrogen, United States). To eliminate any genomic DNA contamination, DNase I (Thermo Fisher Scientific, United States)) was employed. The isolated RNA was subsequently reverse transcribed into complementary DNA (cDNA) using the PrimeScript™ RT reagent Kit (Takara, Japan). For the real-time quantitative PCR, we utilized specific primers along with SYBR^®^ Green PCR Master Mix (Applied Biosystems, United States). The PCR reactions were carried out using a CFX96 instrument equipped with a fluorescence detection system (Bio-Rad, United States). We assessed alterations in fluorescence signals to quantify the expression levels of the target gene. The relative expression levels were determined using the 2^−^ΔΔCt^ method, with β-actin serving as the reference gene.

## 3 Results

### 3.1 The result of bayesian colocalization analysis

Colocalisation analysis indicated shared genetic variants between PD and OP, with a posterior probability of colocalisation (PPH4) of 0.967. In particular, rs3796661was identified as a common genetic variant.

### 3.2 Identification of DEGs in PMO, PD, and ERGs datasets

**FIGURE 2:**
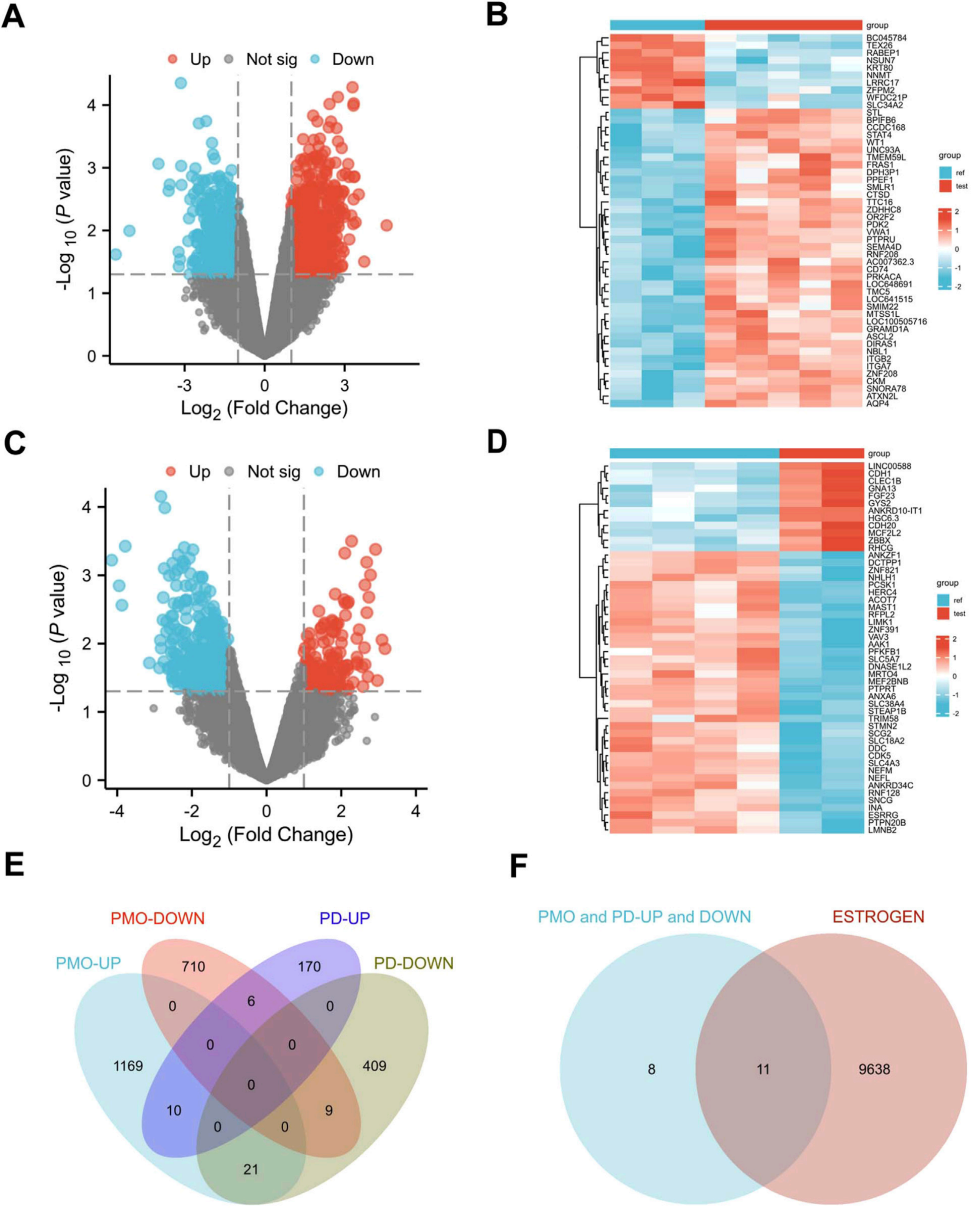
Identification of DEGs in PMO and PD. **(A, B)** Volcano plots and heatmaps for dataset GSE35956; **(C, D)** Volcano plots and heatmaps for dataset GSE20164; **(E)** Venn diagram showing the DEGs shared between PMO and PD; **(F)** Venn diagram illustrating the DEGs in PMO and PD in relation to estrogen.

**FIGURE 3:**
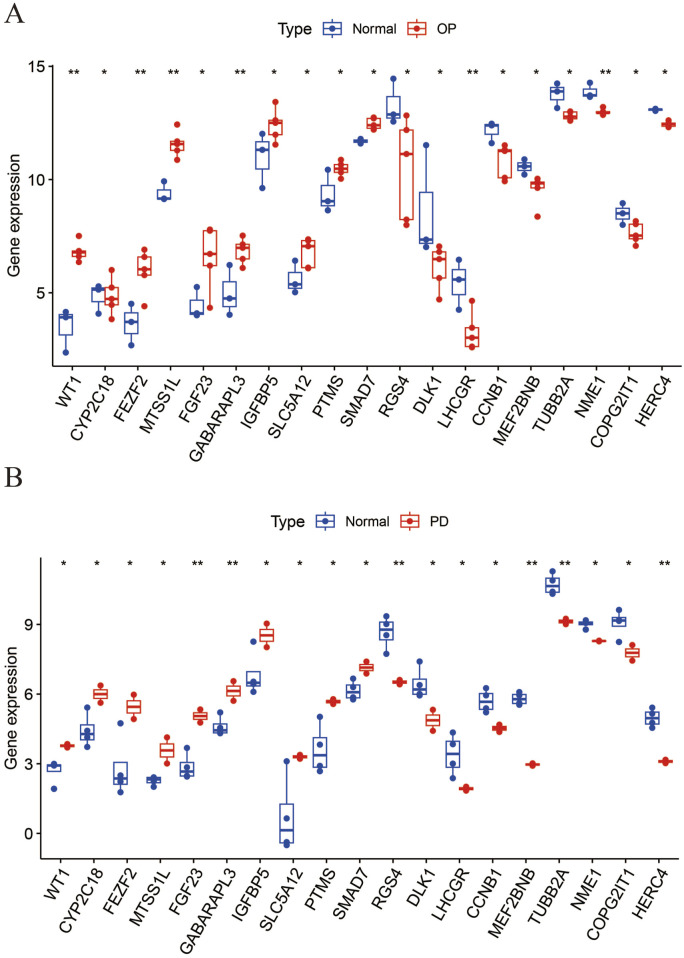
The boxplot illustrates the expression levels of 19 common DEGs between OP and PD. **(A)** OP; **(B)** PD.

We applied a screening threshold of *p* < 0.05 and |log2 FC| > 1.0, identifying 1,879 DEGs in the PMO dataset—1,169 upregulated and 710 downregulated—and 579 DEGs in the PD dataset—170 upregulated and 409 downregulated. The DEGs are illustrated in Figures 2A–D, which use volcano plots and heatmaps to effectively display the upregulated and downregulated genes. Additionally, Figure 2E presents a Venn diagram showing the intersection of 10 co-upregulated and 9 co-downregulated DEGs between the PMO and PD datasets, totaling 19 genes. Figure 3 presents the differential expression of 19 shared DEGs in OP and PD using boxplots. These common genes were compared with ERGs datasets, revealing 11 shared DEGs (Figure 2F).

### 3.3 GO and KEGG pathway enrichment analysis of common DEGs

**FIGURE 4:**
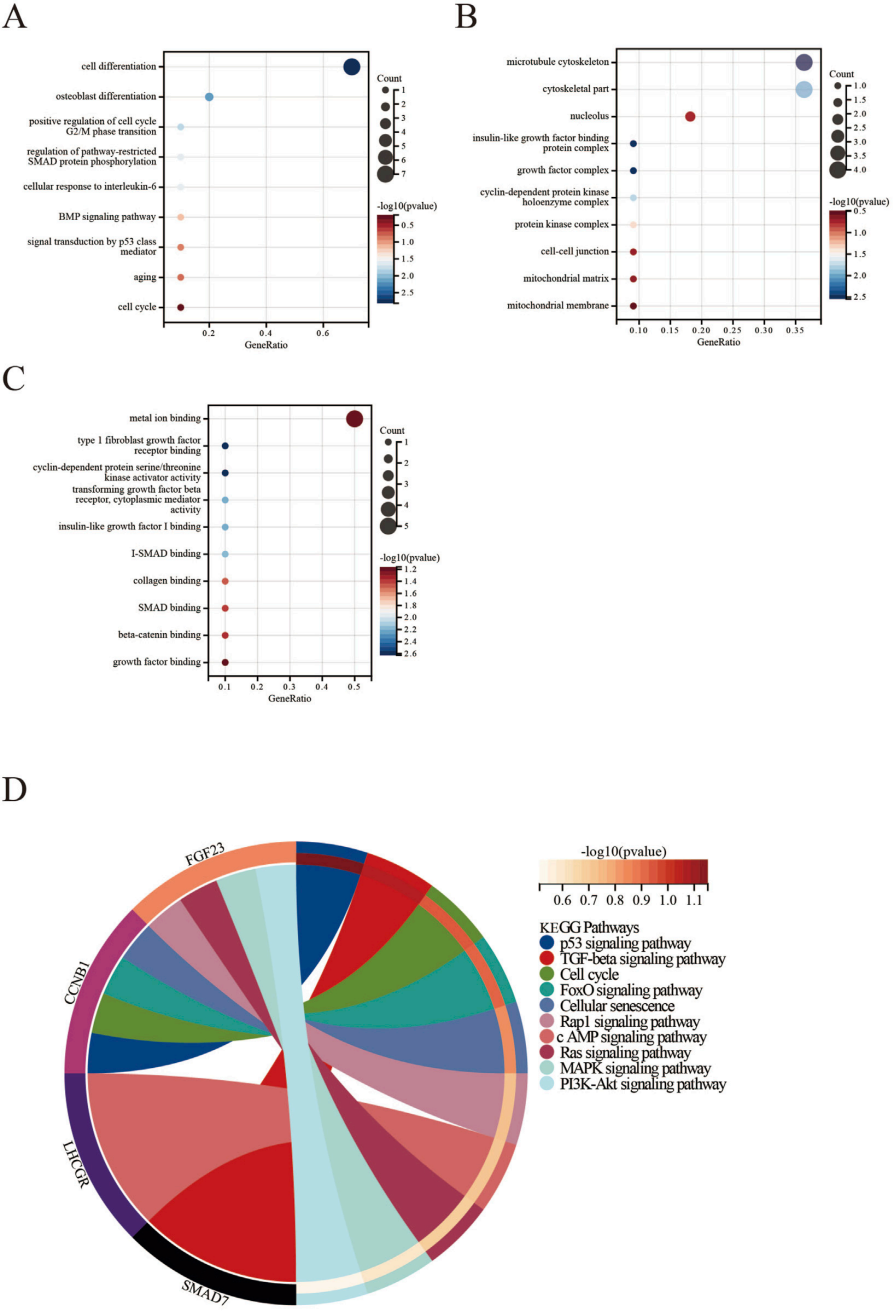
Functional enrichment analysis of DEGs in PMO, PD, and ERGs datasets. **(A)** Biological Processes; **(B)** Cellular Components; **(C)** Molecular Functions; **(D)** KEGG pathways.

We conducted GO and KEGG pathway enrichment analyses of the identified DEGs using the Sangerbox platform. In BP, the DEGs were significantly enriched in several processes, including cell proliferation and osteoblast differentiation. They also showed enrichment in the positive regulation of the G2/M transition of the mitotic cell cycle, pathway-restricted SMAD protein phosphorylation, cellular response to interleukin-6, BMP signaling pathway, signal transduction by p53 class mediator, aging, and the cell cycle (Figure 4A).

For CC, the DEGs were primarily linked to the microtubule cytoskeleton, cytoskeletal parts, nucleolus, insulin-like growth factor binding protein complex, growth factor complex, cyclin-dependent protein kinase holoenzyme complex, protein kinase complex, cell-cell adherens junction, and the mitochondrial matrix and membrane (Figure 4B).

Regarding MF, the DEGs were enriched in several activities, including metal ion binding and type 1 fibroblast growth factor receptor binding. They also exhibited cyclin-dependent protein serine/threonine kinase activator activity, transforming growth factor beta receptor activity, and cytoplasmic mediator activity. Additionally, they were linked to insulin-like growth factor I binding, I-SMAD binding, collagen binding, SMAD binding, beta-catenin binding, and growth factor receptor binding (Figure 4C).

The KEGG pathway analysis revealed significant enrichment of DEGs in several pathways, including the p53 signaling pathway, TGF-beta signaling pathway, cell cycle, FoxO signaling pathway, cellular senescence, Rap1 signaling pathway, cAMP signaling pathway, Ras signaling pathway, MAPK signaling pathway, and PI3K-Akt signaling pathway (Figure 4D).

### 3.4 Construction of the PPI network and identification of hub genes

**FIGURE 5:**
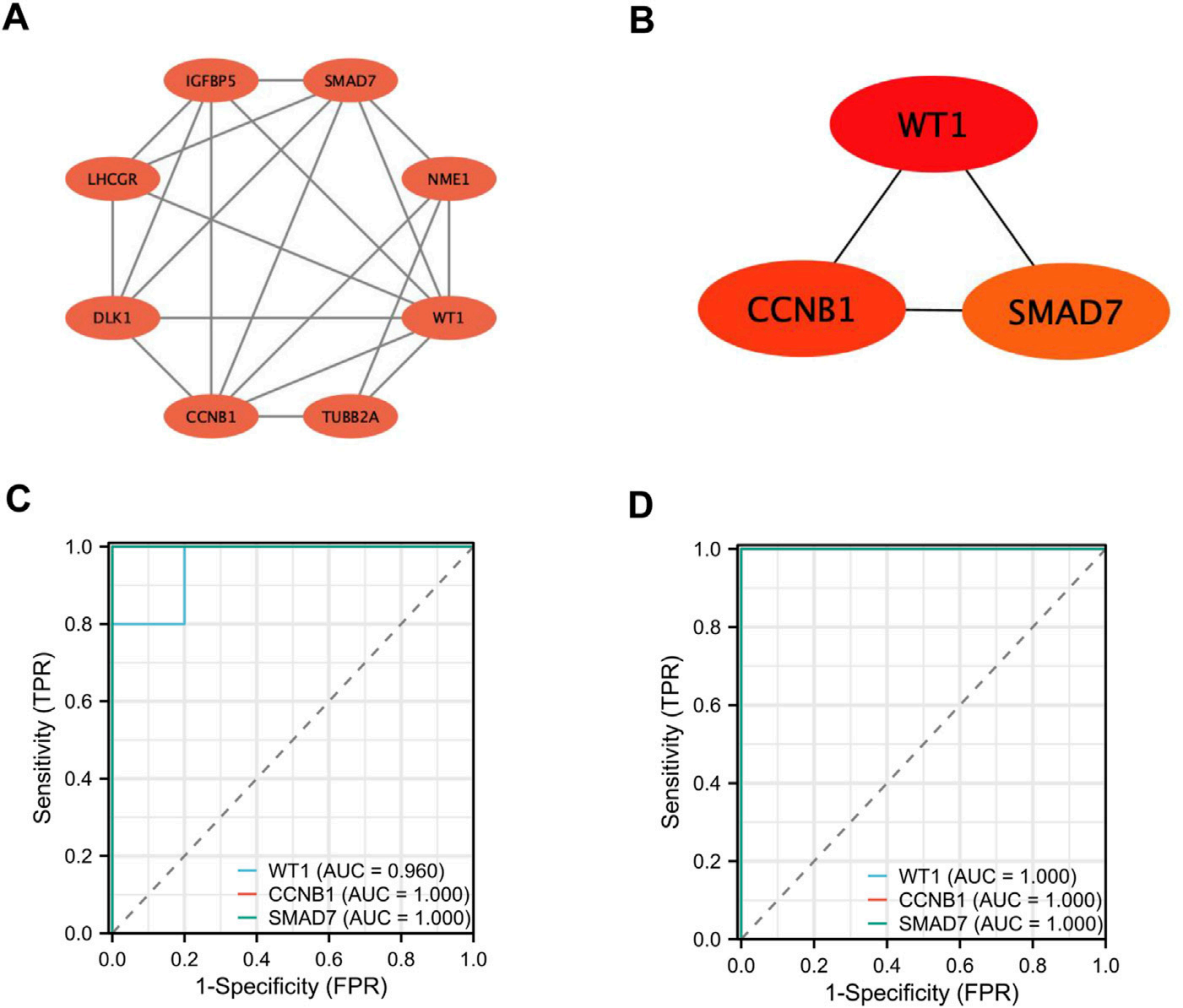
Screening and Identification of Hub Genes. **(A, B)** Construction of the PPI network and identification of hub genes; **(C–D)** Validation of hub gene expression and assessment of diagnostic value.

The PPI network of the DEGs was constructed using the STRING database. It was then visualized with Cytoscape software (Figure 5A). Key hub genes were identified using the CytoHubba plugin in Cytoscape. These hub genes were selected based on their Degree, Closeness, and Betweenness, with those above the median being chosen as the final hub genes. Three potential hub genes were identified (Figure 5B): WT1 transcription factor (WT1), cyclin B1 (CCNB1), and SMAD family member 7 ( SMAD7) (Table 2).

**TABLE 2:** The hub genes based on their degree, closeness, betweenness.

Genes	Degree (score)	Closeness (score)	Betweenness (score)
WT1	7	7	7.5
CCNB1	6	6.5	4
SMAD7	6	6.5	2.83,333,333
DLK1	5	6	0.66,666,667
IGFBP5	5	6	0.5
LHCGR	4	5.5	0.5
NME1	4	5.5	0
TUBB2A	3	5	0
Median value	5	6	2

### 3.5 The diagnostic value of hub gene

We plotted Receiver Operating Characteristic (ROC) curves and calculated the Area Under the Curve (AUC) to assess the diagnostic value of these genes (Figures 5C, D). In the GSE35956 dataset, the AUC values were as follows: WT1 (AUC = 0.960), CCNB1 (AUC = 1.000), and SMAD7 (AUC = 1.000). In the GSE20164 dataset, the AUC values for the genes were as follows: WT1 (AUC = 1.000), CCNB1 (AUC = 1.000), and SMAD7 (AUC = 1.000). The findings indicate that WT1, CCNB1, and SMAD7 could be valuable biomarkers for diagnosing PMO and PD.

#### 3.5.1 TF-gene interaction

**FIGURE 6:**
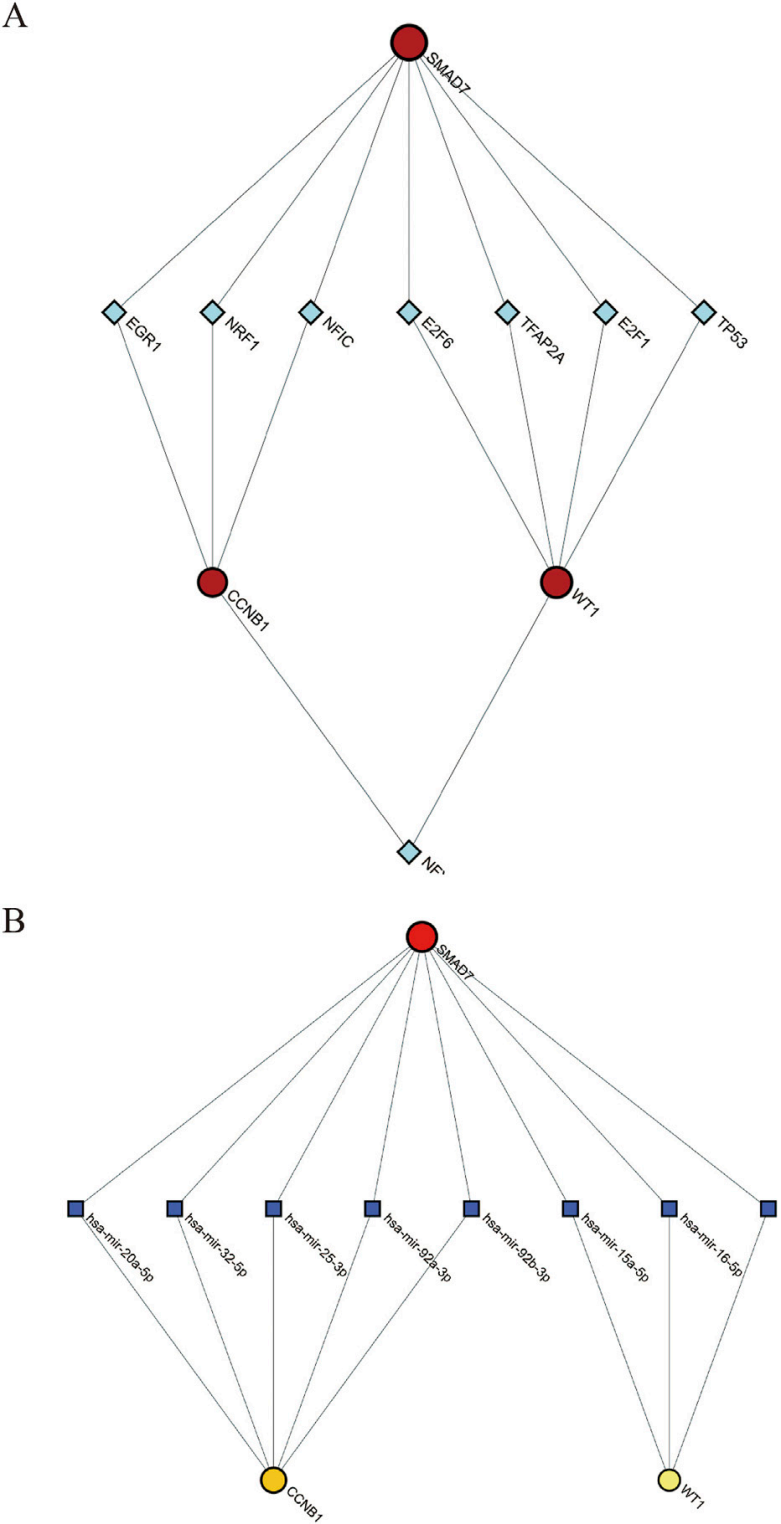
Interaction Networks for TF-Gene and TF-miRNA with Hub Genes. **(A)** TF-Gene interaction network; **(B)** TF-miRNA interaction network. Red circles denote hub genes. Green diamonds represent TFs. Blue squares denote miRNAs.

We predicted and visualized the interactions between transcription factors (TFs) and hub genes using the NetworkAnalyst database. The resulting interaction network included 26 nodes, 27 edges, and 3 seed genes (Figure 6A). The TFs identified for the hub genes are based on a degree cutoff of 1.0 and a betweenness cutoff of 1.0. These TFs include NFIC, NFYA, E2F1, TFAP2A, E2F6, TP53, NRF1, and EGR1 (Table 3). However, these findings require further experimental validation.

**TABLE 3:** The TF-gene interactions of the hub genes.

Id	Name	Degree	Betweeness
4,782	NFIC	2	19.48
4,800	NFYA	2	33
1869	E2F1	2	14.14
7,020	TFAP2A	2	14.14
1876	E2F6	2	14.14
7,157	TP53	2	14.14
4,899	NRF1	2	19.48
1958	EGR1	2	19.48

#### 3.5.2 TF-miRNA coregulatory network

The transcription factor (TF) and microRNA (miRNA) coregulatory network was constructed using the NetworkAnalyst tool. This network comprises 3 hub genes, 33 miRNAs, and 36 TFs. In total, it contains seventy-four nodes and seventy-five edges (Figure 6B). Based on degree and betweenness centrality (Degree cutoff: 1.0, Betweenness cutoff: 1.0), the following miRNAs are associated with the hub genes: hsa-miR-15a-5p, hsa-miR-16-5p, hsa-miR-20a-5p, hsa-miR-25-3p, hsa-miR-32-5p, and hsa-miR-92a-3p (Table 4).

**TABLE 4:** TF-miRNA coregulatory network for hub genes.

Id	Name	Degree	Betweeness
MIMAT0000068	hsa-mir-15a-5p	2	2.3
MIMAT0000069	hsa-mir-16-5p	2	2.3
MIMAT0000075	hsa-mir-20a-5p	2	1
MIMAT0000081	hsa-mir-25-3p	2	1
MIMAT0000090	hsa-mir-32-5p	2	1
MIMAT0000092	hsa-mir-92a-3p	2	1

### 3.6 Interaction of hub genes with diseases

**FIGURE 7:**
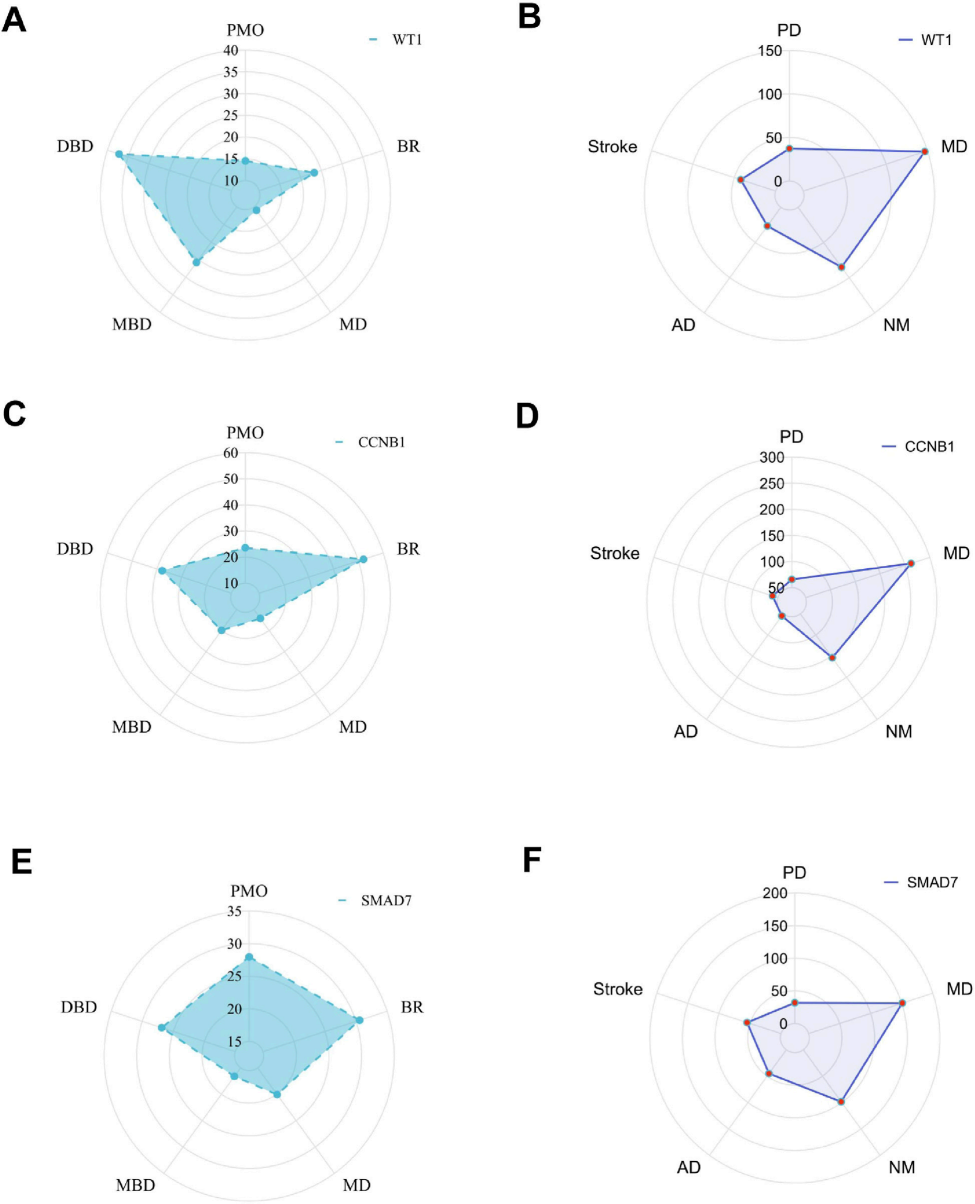
The radar chart. Hub gene interaction with musculoskeletal diseases **(A, C, E)**; Hub gene interaction with nervous system diseases **(B, D, F)**.

We previously identified three hub genes—WT1, CCNB1, and SMAD7—associated with PMO and PD, each showing different inference scores (Table 5, 6). These genes are linked to PMO, bone resorption, musculoskeletal diseases, metabolic bone diseases, and developmental bone diseases, each with varying levels of association. Our results indicate that SMAD7 shows the strongest association with PMO, followed by CCNB1. Regarding bone resorption, CCNB1 is the most closely associated gene, followed by SMAD7. SMAD7 shows the strongest association with musculoskeletal diseases, with CCNB1 in second place. For both metabolic and developmental bone diseases, CCNB1 exhibits the strongest association. Based on these findings, SMAD7 and CCNB1 appear to be the genes most closely related to estrogen-associated PMO (Figure 7). SMAD7 has the strongest association with PD, followed by WT1. In relation to Memory Disorders (MD), CCNB1 is the most closely related gene, followed by SMAD7. SMAD7 also demonstrates the strongest association with Neurobehavioral Disorders (NM), Alzheimer Disease (AD), and Stroke, while CCNB1 ranks second.

**TABLE 5:** Inference scores of hub gene interaction with musculoskeletal diseases.

Gene/diease	PMO	BR	MD	MBD	DBD
WT1	14.54	23.30	10.98	25.82	37.19
CCNB1	23.57	52.07	14.27	19.90	37.90
SMAD7	27.98	30.58	20.07	16.62	26.86

**TABLE 6:** Inference scores of hub gene interaction with nervous system diseases.

Gene/diease	PD	MD	NM	AD	Stroke
WT1	37.29	146.82	85.10	26.55	42.13
CCNB1	65.75	262.33	153.90	54.77	61.09
SMAD7	31.79	150.97	98.57	45.13	54.69

### 3.7 Validation using qRT-PCR

**FIGURE 8:**
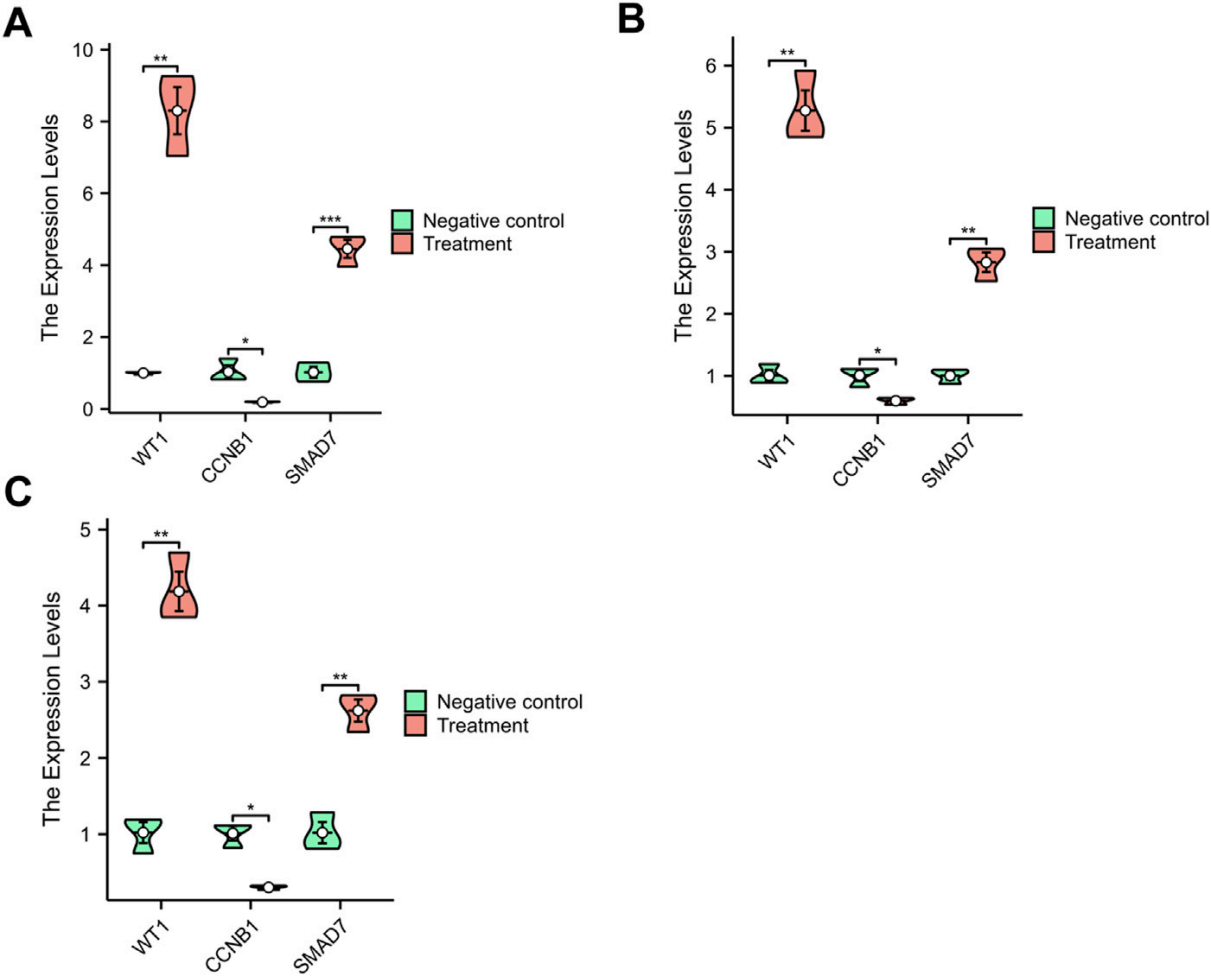
The qRT-PCR Results. **(A)** M3T3-E1 Cells; **(B)** BMSCs; **(C)** PC12 Cells. *p < 0.05, **p < 0.01, ***p < 0.001.

Research shows that the onset of PMO is closely associated with increased apoptosis in osteoblasts (Hu W. X. et al., 2020) and accelerated senescence in bone marrow stromal cells (BMSCs). (Liang et al., 2024). Similarly, the pathogenesis of PD is linked to increased apoptosis in nerve cells (Zhou et al., 2019). To validate the core genes identified in this study, we used cell models to induce apoptosis in both osteoblasts and PC12 cells (Figures 8A, C), as well as senescence in BMSCs (Figure 8B). We assessed the expression levels of the core genes in these models. The qRT-PCR results showed that WT1 and SMAD7 were upregulated, while CCNB1 was downregulated in both cellular models. These findings are consistent with the data from the datasets GSE35956 and GSE20164, confirming the reliability and representativeness of the selected core genes.

## 4 Discussion

In the last 10 years, a notable correlation has been identified between estrogens and the development of several health issues, particularly PMO and PD (Nathan et al., 2012). Women in the postmenopausal phase are particularly susceptible to osteoporosis owing to their diminished estrogen levels (Liu et al., 2014). It is estimated that approximately 50% of females aged over 50 will experience fractures linked to osteoporosis (Yang et al., 2022). Decreased levels of estrogen enhance the activity of osteoclasts, which in turn accelerates the process of bone resorption, resulting in a loss of bone tissue (Andrade et al., 2017).

A deficiency in estrogen leads to modifications in the expression of several cytokines, including tumor necrosis factor-alpha (TNF-α), interleukin-1 (IL-1), and interleukin-6 (IL-6). Increased concentrations of these cytokines facilitate osteoclastogenesis and intensify the process of bone resorption (Iantomasi et al., 2023).

In the current investigation, three distinct groups of DEGs were recognized, which exhibited substantial enrichment in various BP. These processes encompass cell proliferation, the differentiation of osteoblasts, modulation of cell death, regulation of SMAD protein phosphorylation restricted by pathways, cellular responses to interleukin-6, the BMP signaling pathway, signal transduction mediated by p53 class proteins, aging, and the cell cycle. Furthermore, the KEGG pathway analysis demonstrated notable enrichment of DEGs across multiple pathways, including the p53, TGF-beta, cell cycle, FoxO, cellular senescence, Rap1, cAMP, Ras, MAPK, and PI3K-Akt signaling pathways. Growing evidence indicates that estrogens could play a protective role against PD. Both epidemiological and clinical research imply that estrogens may operate as anti-apoptotic agents, antioxidants, or neurotrophic factors, thereby modulating PD and enhancing their interaction with neurotrophic factors (Shulman, 2002). Research involving animal models demonstrates that estrogens affect the synthesis, release, and metabolism of dopamine (Saunders-Pullman, 2003). Importantly, postmenopausal women exhibiting diminished bone mineral density (BMD) are at an elevated risk for developing PD, a risk that can be mitigated through the use of anti-osteoporosis medications (AOMs) (Park K. Y. et al., 2023). These observations suggest a potential link between PMO and PD, indicating that certain genes may play crucial regulatory roles. The analysis identified three groups of DEGs that were significantly enriched in biological processes, including cellular responses to reactive oxygen species, aging, apoptotic signaling pathways, and programmed cell death. These findings imply that the identified genes are intricately connected to the pathogenesis of PD. The study identified rs3796661 as a common genetic variant between OP and PD, suggesting a genetic link between the two conditions. rs3796661 is an intronic variant of the SNCA gene located on chromosome 4q22.1. Abnormal accumulation of α-synuclein is a pathological hallmark of PD. This protein normally regulates synaptic vesicle trafficking and subsequent neurotransmitter release in neurons (Wong and Krainc, 2017). The SNCA gene encoding α-synuclein was identified as the first risk locus for PD in a large-scale GWAS (Simón-Sánchez et al., 2009). In addition, studies outside the central nervous system have shown that SNCA is highly expressed in mature red blood cells, with protein levels remaining elevated in both bone marrow and circulating mature red blood cells (Nakai et al., 2007). Red blood cells derived from Snca−/− bone marrow have lower levels of reactive oxygen species (ROS) and oxidative stress (Renella et al., 2014). Increased oxidative stress has been shown to promote bone loss induced by ovariectomy, suggesting that Snca is a key regulator of bone homeostasis. Its absence results in a 40% reduction in the magnitude of estrogen deficiency-induced bone loss (Calabrese et al., 2016).

Research has demonstrated that insufficient levels of estrogen can lead to the disruption of specific microRNAs (miRNAs) that play a critical role in bone metabolism. The dysregulation of these miRNAs has a significant impact on the process of bone remodeling by modifying gene expression and influencing various signaling pathways (Iantomasi et al., 2023). For instance, miR-21-5p has been identified as a target of SKP2, which is instrumental in the regulation of osteoclast differentiation (Huang et al., 2021). In patients diagnosed with PMO, the expression of miR-146a is notably reduced, which in turn affects bone metabolism through its modulation of the Wnt/β-catenin signaling pathway (Liu et al., 2021). Furthermore, this investigation identified several miRNAs that are closely linked to hub genes, including hsa-mir-15a-5p (He et al., 2021), hsa-mir-16-5p (Wang et al., 2023), hsa-mir-25-3p (Feng et al., 2022), hsa-mir-32-5p (Wang H. J. et al., 2021), and hsa-mir-92a-3p (Zheng et al., 2024) with a particular emphasis on their relationship with SMAD7.

TFs play a key role in regulating the transcription of target genes, making their levels useful for identifying potential biomarkers. This study identified relevant TFs as regulators of DEGs associated with the pathogenesis of PMO and PD through the TF-mRNA interaction network. NFIC is a critical transcription factor in organ development and stem cell biology, enhancing the reprogramming efficiency of pluripotent stem cells induced by mineralised tissues and maintaining the stem cell niche in mineralised tissues (Lee et al., 2022). Studies have also identified NFIC as an important regulatory transcription factor in neurodegenerative processes (Rahman et al., 2019). NFYA plays a role in the regulation of neurodegenerative diseases and haematopoietic stem cells (Bungartz et al., 2012; Henderson et al., 2021). E2F1 regulates the cell populations essential for bone repair and plays a distinct role in bone formation and repair (Premnath et al., 2024). E2F1 has also been shown to alter cell viability, induce neurotoxicity and have implications in PD (Xu et al., 2005). E2F6 counteracts the pro-apoptotic activity of E2F1 (Kikuchi et al., 2007). TFAP2A affects neuronal function and survival and may play a role in the pathogenesis of neurodegenerative diseases (Hu Y. et al., 2020). TFAP2A is also involved in osteoblast activity (Chen et al., 2018). TP53 can interfere with normal angiogenesis, glycolysis and apoptosis and may play a role in the pathogenesis of osteoporosis and Parkinson’s disease (Ge et al., 2021; James et al., 2024). NRF1 has been identified as a trans-activator of human dopamine transporters, the dysfunction of which is associated with psychiatric and neuronal disorders (Zhao et al., 2019). NRF1 has been found to play a distinct role in the regulation of antioxidant enzymes during inflammation-induced oxidative stress in osteoblasts (Park et al., 2016). EGR1 inhibits the downregulation of RANKL and sclerostin gene expression, resulting in reduced levels of these proteins in osteoblasts (Miura et al., 2024). EGR1 has also been implicated in the neuroprotection of motor function and dopaminergic neurons in Parkinson’s disease (Guo et al., 2023).

Unlike TFs, microRNAs act post-transcriptionally to regulate gene expression. They are single-stranded RNAs that bind to target mRNAs, causing cleavage and a reduction in expression. These miRNAs offer several advantages as non-invasive biomarkers detectable in body fluids such as urine and saliva, making them promising for biomarker development. While hsa-miR-15a-5p has not been reported in PMO, it is upregulated in multiple myeloma, another bone disease, and is also a biomarker of ageing and cognitive impairment in the elderly (Li et al., 2020; Ye et al., 2020). hsa-miR-16-5p serves as a potential biomarker for predicting duloxetine response in major depressive disorder, suggesting its role in neurological disorders (Kim et al., 2019). Studies also suggest that hsa-miR-16-5p is associated with osteoclast activity, with its expression levels potentially reflecting the degree of bone resorption (Qin et al., 2016). hsa-miR-20a-5p, an NHL-associated miRNA within the hsa-miR-17–92 cluster, regulates B-cell development and central tolerance, but its links to PMO and PD remain unexplored (Fernandes et al., 2022). Research has shown an association between hsa-miR-25-3p and postmenopausal femoral neck BMD (Faraldi et al., 2024). hsa-miR-25-3p has been found to affect biological pathways associated with neuropsychiatric disorders (Van der Auwera et al., 2022). No studies have investigated the relationship between hsa-miR-92a-3p and PMO or PD.

SMAD7 serves a crucial inhibitory function within the TGF-β signaling pathway, categorizing it as a member of the inhibitory Smad (I-Smad) family. Its mechanism of action involves binding to the TGF-β receptor, which effectively obstructs the phosphorylation processes of Smad2 and Smad3. The role of SMAD7 extends across a variety of physiological activities, notably in regulating cellular proliferation, differentiation, and apoptosis (Yan et al., 2009). Recent studies suggest a potential correlation between SMAD7 and conditions such as PMO and PD (Yano et al., 2012; Karampetsou et al., 2022). Notably, the persistent expression of Smad7 in osteoblasts has been shown to significantly diminish both the proliferation and differentiation of these cells (Yano et al., 2012). Furthermore, bone marrow stromal cells (BMSCs) derived from Smad7 knockout (KO) mice exhibited a diminished capacity for bone formation, coupled with an enhanced propensity for adipogenesis and osteoclast differentiation (Li et al., 2014). Investigations have revealed that estrogen contributes to bone protection, in part through the modulation of TGF-β signaling, a pathway integral to the understanding of osteoporosis progression. The reduction of estrogen levels post-menopause may consequently impact the expression or functional activity of SMAD7, thereby exacerbating the progression of osteoporosis (Hioki et al., 2022). Additionally, the estrogen-related receptor β (ESRRB) has been identified as a transcriptional activator of SMAD7, serving to inhibit the phosphorylation of SMAD2/3 and its subsequent nuclear translocation (Li and Zheng, 2023). Moreover, emerging evidence suggests a potential linkage between the neurodegenerative processes underlying PD and the TGF-β signaling pathway (Karampetsou et al., 2022). Consequently, SMAD7 presents as a promising target for therapeutic interventions aimed at addressing postmenopausal osteoporosis and Parkinson’s disease.

CCNB1, or Cyclin B1, serves as a pivotal regulatory protein within the cell cycle, predominantly exerting its influence during the G2/M transition, thereby governing the process of cell mitosis (Chang et al., 2016). In osteocytes, CCNB1 collaborates with cyclin-dependent kinase 1 (CDK1) to facilitate the progression of cells into mitosis (Sun et al., 2019). A deficiency in CCNB1 within the context of PMO has been shown to diminish osteoblast proliferation and hinder the formation of new bone (Li et al., 2024). Furthermore, CCNB1 is also crucial in modulating the cell cycle in neural cells (Yan and Ziff, 1995). Consequently, enhancing the expression of CCNB1 or refining cell cycle regulation may bolster the proliferation of both osteoblasts and neuronal cells, thereby fostering the recovery of bone mass, decelerating the onset of osteoporosis, and alleviating the manifestations of PD.

The Wilms’ Tumor 1 (WT1) gene serves primarily as a tumor suppressor, with its encoded protein playing a crucial role in the regulation of cellular processes such as proliferation, differentiation, and apoptosis. In the context of PMO, the aberrant overexpression of WT1 has been associated with osteoblast apoptosis, resulting in reduced bone formation, heightened bone loss, and an exacerbation of disease progression (Wang C. et al., 2021). Notably, elevated levels of WT1 protein are found in osteoclast precursors; however, this expression significantly diminishes as osteoclastogenesis occurs. Additionally, increased expression of WT1 antisense RNA has been shown to facilitate osteoclastogenesis and enhance the differentiation stability of osteoclasts (Li et al., 2016). Furthermore, WT1 is integral to the differentiation processes of nerve cells (Sarfstein and Werner, 2006). In neuronal contexts, elevated WT1 levels correlate with the promotion of neuronal apoptosis, whereas diminished WT1 levels are associated with its suppression (Liu et al., 2001).

The gene-disease radial plot visually represents how genes are expressed or associated with different diseases. The shape of the polygon, including its peaks and valleys, shows unique gene expression patterns across various diseases. This radar plot displays the expression patterns of the genes WT1, CCNB1, and SMAD7 in relation to PMO and PD. Inference scores reveal that SMAD7 is more strongly associated with PMO and MD, whereas CCNB1 shows a closer association with BR, MBD, and DBD. SMAD7 exhibits the strongest association with PD, NM, AD, and Stroke. In relation to Memory Disorders (MD), CCNB1 is the most closely related gene.

In summary, we initially identified 11 DEGs by comparing samples from PMO, PD, and GREs. Subsequent PPI analysis allowed us to narrow this list down to 3 hub genes, which were then validated through ROC. Functional enrichment analysis indicated that these genes play a role in several important pathways. Specifically, they are involved in the regulation of the p53 signaling pathway, the TGF-beta signaling pathway, the cell cycle, and the FoxO signaling pathway.

This study presents several important limitations. One limitation of this study is the small sample size in both the training and validation sets. Future studies should use larger sample sizes to improve result reliability. Secondly, the study lacked prognostic analysis due to the GEO database’s limited patient information and prognostic data. This limitation hinders a deeper examination of patient outcomes. Additionally, while we identified hub genes with promising diagnostic potential for PMO and PD, the specific mechanisms of these genes in different datasets are still unclear.

## Data Availability

The datasets presented in this study can be found in online repositories. The names of the repository/repositories and accession number(s) can be found in the article/supplementary material.

## Abbreviations

PMO, Postmenopausal osteoporosis; PD, Parkinson’s disease; ER, Estrogen receptor; E2, Estradiol; DEGs, Differentially expressed genes; CTD, Toxicogenomics Database; KEGG, Kyto encyclpedia of Genes and Genomes; BMD, Bone Mineral Density Bone Mineral Density.
